# Resolving the Effect of Oxygen Vacancies on Co Nanostructures Using Soft XAS/X-PEEM

**DOI:** 10.1021/acscatal.2c00611

**Published:** 2022-07-14

**Authors:** Chengwu Qiu, Yaroslav Odarchenko, Qingwei Meng, Shaojun Xu, Ines Lezcano-Gonzalez, Paul Olalde-Velasco, Francesco Maccherozzi, Laura Zanetti-Domingues, Marisa Martin-Fernandez, Andrew M. Beale

**Affiliations:** † Department of Chemistry, University College London, 20 Gordon Street, London WC1H 0AJ, U.K.; ‡ Research Complex at Harwell (RCaH), Harwell, Didcot OX11 0FA, Oxfordshire, U.K.; § School of Chemical Engineering and Light Industry, Guangdong University of Technology, Guangzhou 510006, China; ∥ Cardiff Catalysis Institute, School of Chemistry, Cardiff University, Cardiff CF10 3AT, U.K.; ⊥ Diamond Light Source, Harwell, Didcot OX11 0DE, Oxfordshire, U.K.

**Keywords:** oxygen vacancies, TiO_2_, cobalt, metal-support interaction, nanoparticle size, XAS/X-PEEM

## Abstract

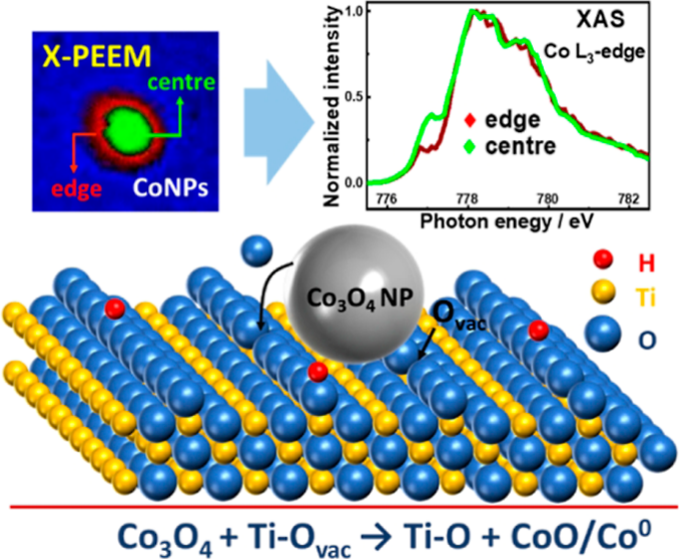

Improving both the extent of metallic Co nanoparticle (Co NP) formation and their stability is necessary to ensure good catalytic performance, particularly for Fischer–Tropsch synthesis (FTS). Here, we observe how the presence of surface oxygen vacancies (O_vac_) on TiO_2_ can readily reduce individual Co_3_O_4_ NPs directly into CoO/Co^0^ in the freshly prepared sample by using a combination of X-ray photoemission electron microscopy (X-PEEM) coupled with soft X-ray absorption spectroscopy. The O_vac_ are particularly good at reducing the edge of the NPs as opposed to their center, leading to smaller particles being more reduced than larger ones. We then show how further reduction (and O_vac_ consumption) is achieved during heating in H_2_/syngas (H_2_ + CO) and reveal that O_vac_ also prevents total reoxidation of Co NPs in syngas, particularly the smallest (∼8 nm) particles, thus maintaining the presence of metallic Co, potentially improving catalyst performance.

## Introduction

Metal nanoparticles (NPs) anchored to a support are widely used as heterogeneous catalysts in a number of important industrial chemical processes.^1,2^ Such heterogeneous catalysts owe their activity to the formation of unique metal–support interactions (MSI), which typically result in materials containing highly dispersed metal species stabilized in a particular electronic or coordination state.^3^ A critical challenge when making them concerns the nature and properties of the NPs be they metal, metal oxide, or, as is often the case, a mixture of both. For the majority of preparation methods used, the NPs in the fresh catalysts are present in the oxide form,^4,5^ so a subsequent activation step is required to obtain metallic NPs.^6^ The intimacy and nature of the NPs’ interaction with the catalyst support, which is useful to ensure that these are well distributed and stabilized, is well known to influence the rate and extent of reduction.^7^ This has been illustrated in many studies, showing that metal oxide NPs are difficult to fully reduce to the metallic phase in comparison with their unsupported metal oxide counterparts.^8,9^ Notably, for reactions that require the presence of metallic NPs as the active component,^10,11^ such as CO_2_ hydrogenation,^12^ Fisher–Tropsch Synthesis (FTS),^4^ selective hydrogenation reactions,^13^ and light-alkane dehydrogenation,^14^ this can be particularly problematic in that either there is an underutilization of the metal leading to lower surface-specific activity or the selectivity to the desired product is adversely affected by the oxidic phase(s). To some extent, the activation treatment can be performed under harsher conditions, although this can lead to NP sintering or solid-state reaction between the NPs and support. Alternative strategies to facilitate the reducibility of metal oxide NPs include limiting the extent of the MSI and the addition of noble metals (Pt, Re, Ru, etc.).^15,16^ A consequence of weakening the MSI however, is the enhanced mobility of the supported NPs, which leads to increased risk of aggregation,^17^ whereas the presence of noble metals has been shown to affect activity and selectivity and also to accelerate deactivation.^18,19^

Supported cobalt NPs are exemplary as heterogeneous catalysts, where the abovementioned phenomena particularly apply. They are perhaps best known as catalysts for FTS, a widely applied method for the production of liquid transportation fuels and high-value chemicals, and are typically characterized by higher activity, higher chain-growth probability, and lower water gas shift activity than iron-based FTS catalysts.^20^ It has generally been shown that metallic Co NPs are the active phase on common supports such as TiO_2_, SiO_2,_ and Al_2_O_3_;^15,20^ cobalt oxide is typically present initially as Co_3_O_4_,^8^ which is then reduced to CoO and eventually Co metal before being used in the reaction.^9,21^ The first step of Co_3_O_4_ to CoO is comparatively easy to effect, while the second step of CoO to Co^0^ is more difficult owing to the interaction between the metal and the support.^9,21^ This is further complicated by the tendency of NPs to agglomerate or else to become encapsulated by reduced TiO_2-x_,^22^ while recent reports have also shown Co NPs tend to spread on TiO_2_ surfaces.^23^ Consequently, alternative synthetic approaches need to be developed so as to enable facile Co NP reduction avoiding the well-documented downsides of the current preparation methods.

Interestingly, it has previously been reported that oxygen vacancies (O_vac_) or the presence of Ti^3+^ on TiO_2_ surfaces—created by thermal annealing or plasma treatment,^24,25^ enhance metal oxide reducibility in TiO_2_ supported catalysts, proposed to occur through the capture of oxygen-containing species via oxygen spillover.^24,26^ Parameters such as support particle size and morphology have been shown to influence O_vac_ formation and reducibility of NPs,^27,28^ however, to date there is not a clear understanding of the promotional effect of O_vac_ as a function of the supported metal (oxide) NP size. This is particularly important for FTS because there is a strong size dependency on catalyst performance and stability,^29,30^ as well as for other well-known structure-sensitive catalytic reactions, such as CO_2_ hydrogenation.^31,32^ In this work, we have applied a combined surface-sensitive spectroscopic and microscopic method which is capable of probing the metal–support interface so as to be able to probe and determine the surface O_vac_ promotional effect on the reduction behavior of supported Co_3_O_4_ NPs. To this end, a two-dimensional (2D) Co/TiO_2_ sample was prepared by depositing on rutile (110) presynthesized Co_3_O_4_ NPs, exhibiting a range (6–18 nm) of particle sizes relevant to FTS. The sample comprises regions with differing concentrations of O_vac_, generated by air plasma treatment^33^ determined using O K-edge and Co L_3_-edge spectroscopy. *Quasiin situ* X-ray photo emission electron microscope (X-PEEM) coupled with soft X-ray absorption spectroscopy (XAS) was then used to directly determine the behavior of these systems under reducing and syngas conditions, enabling us to correlate the impact of O_vac_ affecting the phase evolution of different sized Co NPs for the first time.

## Results and Discussion

### Correlating the Size and Oxidation State of Co NPs

Two regions were identified by XAS/X-PEEM which differed in the number of O_vac_ as determined from the O K-edge spectra (vide infra). These are labeled Co/Ti-1 (high concentration of O_vac_) and Co/Ti-2 (low concentration of O_vac_), with these differences attributed to the indiscriminate ability of air/O_2_ plasma treatment to create O_vac_.^25^ The two regions are then further divided into subregions A and B, as it was not always possible to locate exactly the same region after each gas treatment.

**Figure 1:**
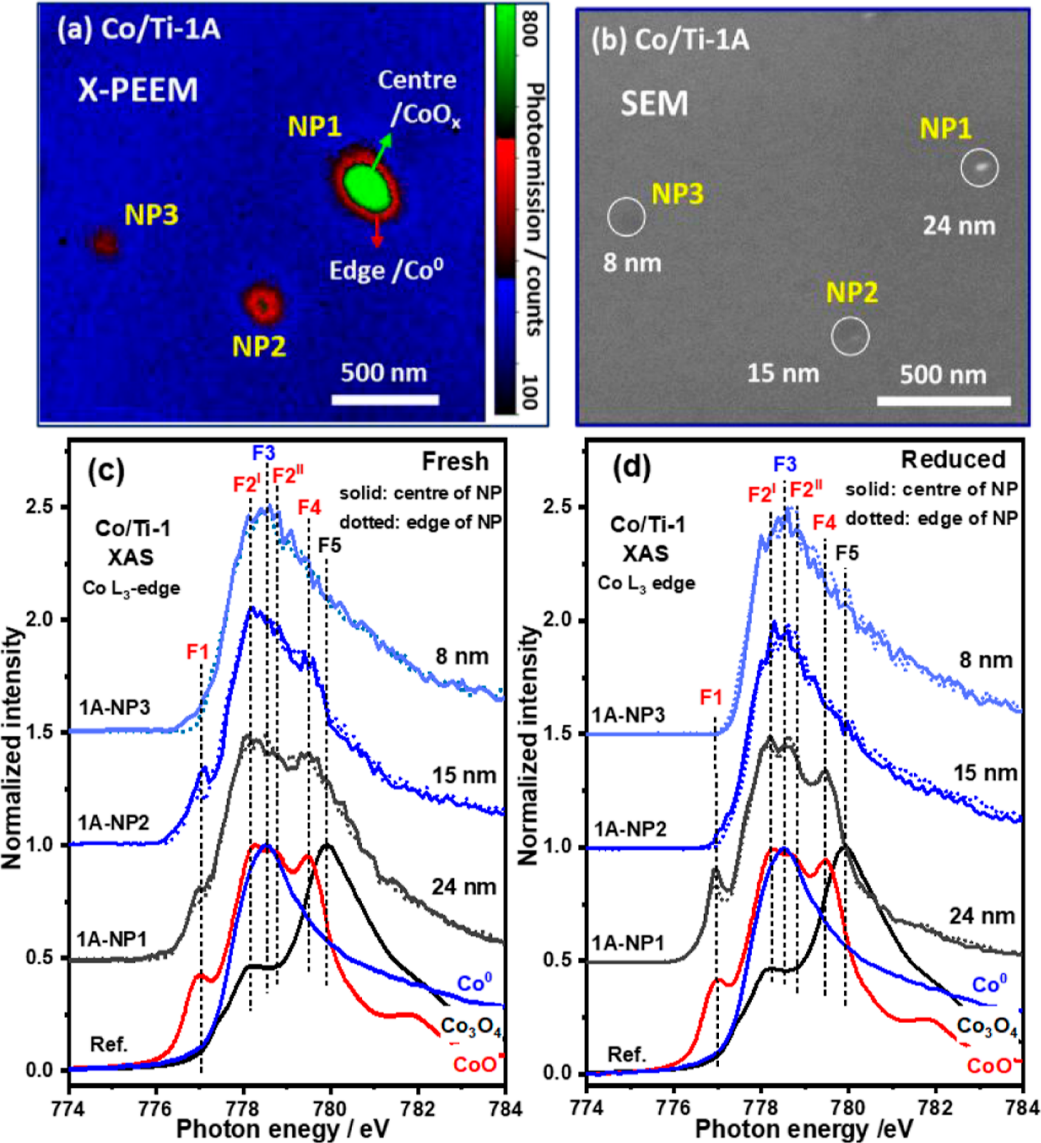
(a) Focused X-PEEM image (false color map, recorded at 778.5 eV) of Co/TiO_2_, in which edges (red) and centers (green) of interested Co NPs are indicated, recorded on a region of the sample determined to have a higher proportion of O_vac_. (b) Corresponding high-resolution SEM image. (c,d) Co L_3_-edge XAS spectra of the NPs before (c) and after (d) reduction. Co_3_O_4_ directly transforms to CoO/Co^0^ in the fresh sample and fully to metallic Co in the NPs <8 nm. Small Co NPs (<15 nm) are fully reduced demonstrating that for all sizes of NPs studied, the edges are easy to reduce. Note that the image in (a) is a portion cropped from the original X-PEEM image shown in Figure S4a. F1 (777.0 eV), F2^I^ (778.2 eV), F2^II^ (778.7 eV), and F4 (779.5 eV) label the principal features in the spectra of CoO; F3 (778.5 eV) corresponds for Co^0^; whilst F5 (779.9 eV) is the main feature consistent with the presence of Co_3_O_4_.

X-PEEM, scanning electron microscopy (SEM), and Co L_3_-edge XAS spectra for one region (labeled as Co/Ti-1) of the Co/TiO_2_ sample featured in this study are shown in Figure 1. The sample was made from presynthesized unsupported spherical Co_3_O_4_ NPs (shown in Figure S1) that were dispersed in ethanol, centrifuged before being dip-coated onto the titania (rutile) substrate, dried, and subjected to air plasma treatment. The Co NPs distribution (interparticle distance > 500 nm) in the pristine Co/TiO_2_ was determined by atomic force microscopy (AFM, Figure S2) to possess a mean NP size of 12.7 ± 7.0 nm. The initial presence of some CoO (instead of Co_3_O_4_) and small amounts of Co^0^ was confirmed by X-ray photoelectron spectroscopy (XPS, Figure S3a). At this point, we hypothesize that partial reduction of Co NPs has more to do with the properties of TiO_2_ rather than the measurement performed on the sample. Subsequently, the sample was loaded into the X-PEEM apparatus via a preparation chamber that allows controlled gas dosing and heat treatment of the sample.^23^ The X-PEEM images (Figures 1a and S4a–d) contain spots with different brightness and diameters, reflecting the differences in the size of the NPs. The absolute size of the Co NPs was confirmed by high-resolution SEM (Figures 1b and S5), revealing the particles to be ∼10 times smaller than the size shown in the X-PEEM images (Figure S4a–f). This difference in observed spatial resolution (∼20 nm) is attributed to the X-ray energy and incidence angle as well as sample nature (conductivity and topography).^34,35^

Co L_3_-edge XAS spectra for the single Co NPs of interest before H_2_ reduction are shown in Figure 1c. Consistent with the XPS data, the cobalt species are revealed by linear combination fitting to contain a mixture of Co_3_O_4_, CoO, and Co^0^. As can be seen in Tables 1 and S1, a number of particles have been analyzed from regions in the sample that have both a higher (Co/Ti-1A) and lower (Co/Ti-2A) number of O_vac_ (vide infra). As a result, we observe that the degree of reduction of the Co NPs in region Co/Ti-1A is greater than in region Co/Ti-2A. However, the extent also depends on the Co NP size. In Co/Ti-1A, the Co NP labeled as 1A-NP1 is ∼ 24 nm in diameter and comprises mostly (∼90%) cobalt oxides, whilst 1A-NP3, closer to 8 nm in size, contains mainly (∼90%) Co^0^ with only minor amounts of both CoO and Co_3_O_4_. In contrast, in the latter region (Co/Ti-2A, Figure S4c), the main component in the big NP (2A-NP2, 23 nm) is determined to be Co_3_O_4_ (∼75%) and little Co^0^ is found. However, the smaller NPs contain more CoO (by dint of a feature at ∼777 eV highlighted as F1 in Figure S7a) the major component throughout all NPs remains the oxides and particularly Co_3_O_4_ (see Table S1). The cobalt oxide that remains in the big NPs is thought to be due to the difficulty in reducing the core particularly after the surface is reduced.^36^

As can be seen from Figure 1 and Table 1, the spatial resolution of the X-PEEM instrument allows for identifying differences in the Co L_3_ XAS fine structure when comparing the spectra at the edge of the sample (cobalt–titania interface) and the center (bulk cobalt) of an NP (see Figures 1a,c and S6). From Figure 1c, the F1 features at the NPs’ edge are clearly weaker than those seen in the center, indicating that cobalt at the edge of the NPs is more reduced; this observation is consistent with the composition data derived from linear combination fitting reported in Table 1. In contrast, differences in the spectra show no spatial dependency in the Co/Ti-2A region (Figure S7a), remaining essentially unchanged.

**Table 1:** Linear Combination Fitting Results from Co L_3_-Edge XAS Spectra of Region Co/Ti-1 (See Fitting Profiles in Figure S8)^a^

spectrum		size/nm	Co^0^/%	CoO/%	Co_3_O_4_/%	*R*-factor	reduced χ-square
fresh	1A-NP1-center	24	9.9(2.4)	62.1(1.7)	28.0(1.6)	0.01464	0.001921
	1A-NP1-edge		11.5(4.5)	58.7(4.1)	29.8(3.0)	0.01094	0.001830
	1A-NP2-center	15	55.6(3.3)	44.4(2.4)	0(0.3)	0.00888	0.000794
	1A-NP2-edge		58.1(2.8)	37.8(2.8)	4.1(4.8)	0.01139	0.001089
	1A-NP3-center	8	82.1(2.5)	12.2(2.5)	5.7(4.5)	0.00978	0.000883
	1A-NP3-edge		90.2(2.7)	4.6(2.7)	5.2(4.7)	0.01133	0.001012
reduced	1A-NP1-center	24	32.2(4.5)	66.8(3.7)	1.0(5.8)	0.01189	0.001745
	1A-NP1-edge		38.9(4.4)	60.6(3.6)	0.5(5.6)	0.01105	0.001618
	1A-NP2-center	15	99.5(2.2)	0(2.2)	0.5(0.3)	0.00841	0.000682
	1A-NP2-edge		100(0)	0(0)	0(0)	0.02485	0.002165
	1A-NP3-center	8	100(0)	0(0)	0(0)	0.02971	0.002593
	1A-NP3-edge		100(0)	0(0)	0(0)	0.02764	0.002518
2							
syngas adsorption	1A-NP1-center	24	43.0(6.9)	57.0(5.1)	0(0.8)	0.02919	0.003290
	1A-NP1-edge		50.8(2.9)	44.9(3.2)	4.3(4.3)	0.01196	0.001178
	1A-NP2-center	15	100(0)	0(0)	0(3.8)	0.03490	0.002633
	1A-NP2-edge		100(0)	0(0)	0(3.8)	0.06598	0.006382
	1A-NP3-center	8	78.9(5.0)	13.6(4.2)	7.5(7.0)	0.01189	0.001691
	1A-NP3-edge		86.8(5.3)	11.8(5.8)	1.4(8.3)	0.01776	0.002723

a The edge and center definitions are shown in Figures 1a and S6. The numbers in parenthesis are fitting errors.

To examine the behavior of the Co NPs under a reducing atmosphere, the sample was treated in pure H_2_ (1 × 10^–6^ mbar) at 623 K for 3 h. In Figure 1d NPs ≤15 nm [1A-NP3(8 nm)/1A-NP2(15 nm)], an initial glance at the shape of the XAS spectra indicates that both essentially contain only Co^0^. However, the bigger NP (1A-NP1, 24 nm) still comprises largely (>60%) CoO (see Table 1). Lastly, the differences in the spectra between the edge and center follow the same trend as seen in the fresh sample, indicating that the Co NP edges are easier to reduce than the centers. For example, feature F1 in the spectra recorded at the edge of 1A-NP1 (24 nm, 38.9% of Co^0^) is lower in intensity than that recorded from the NP center (32.2% of Co^0^). In contrast, for the Co/Ti-2 region which contains a lower [O_vac_], particularly the portion of Co/Ti-2B where no O_vac_ are observed, the Co NPs prove to be difficult to reduce even at 623 K in H_2_ (Figure S7b). We observe again a significant difference in the XAS spectra at the edges and centers of the NPs in this sample after reduction; notably, the F1 intensity of the spectra at the edges due to the presence of cobalt oxides are always greater than those recorded at the centers. This suggests that unlike the Co NPs in the presence of more [O_vac_], the centers of the Co NPs are easier to reduce in these regions (see compositional differences in Tables 1 and S1).

### Determining the Presence of Oxygen Vacancies on the TiO_2_ Surface

**Figure 2:**
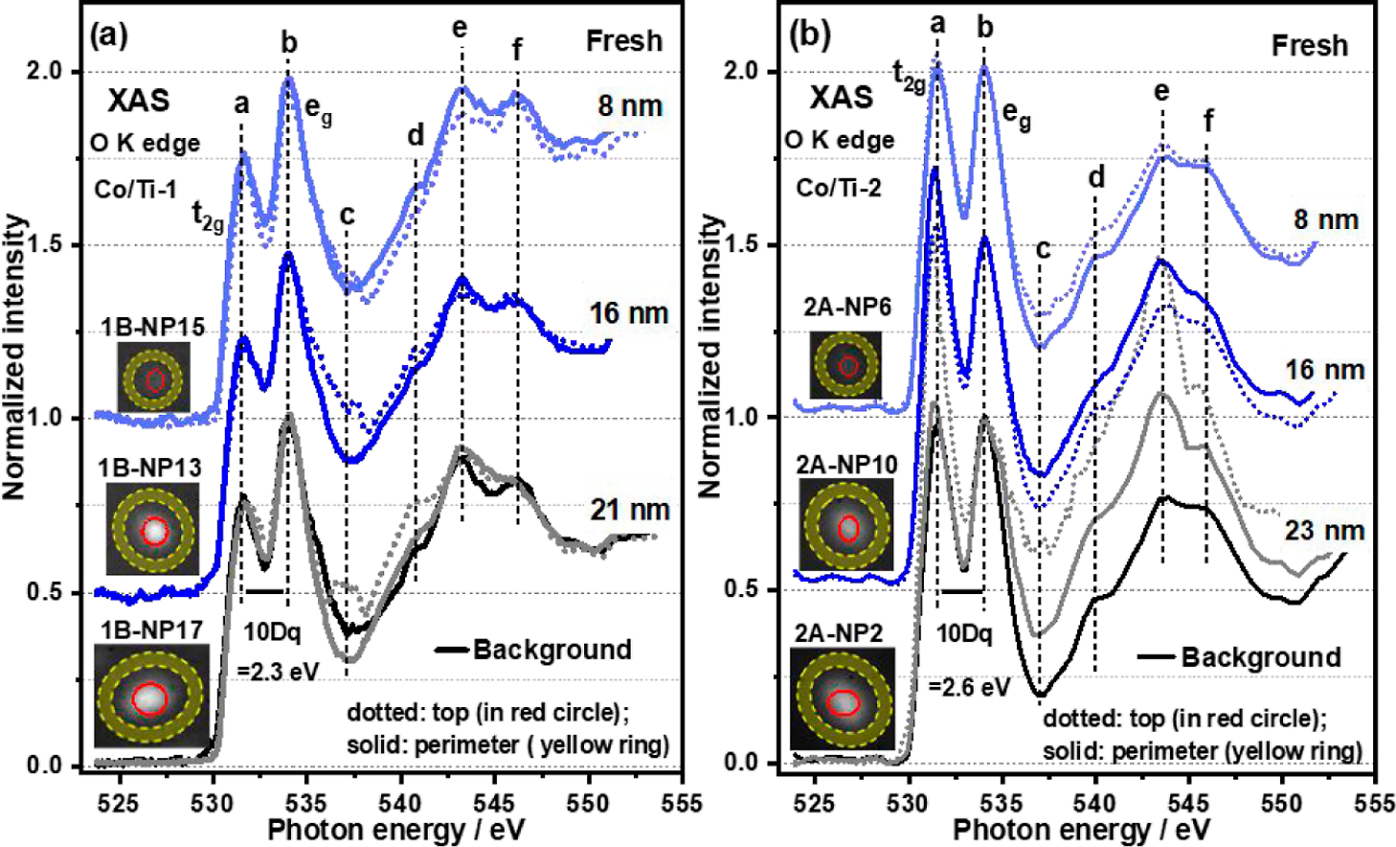
Local XAS spectra of O K-edge in the defined periphery (shaded yellow ring) and top (red ring) of the NPs from regions Co/Ti-1 (a) and Co/Ti-2 (b). All the spectra in (a,b) are normalized to the maximum of the e_g_ peaks. Note the strong feature c (dotted line) is attributed to the contribution of cobalt oxides.

In order to understand if it is possible to correlate the behavior of the NPs with the properties of the TiO_2_ support, spatially resolved O K-edge XAS spectra at the center and periphery of the Co NPs were recorded and shown in Figures 2, S4, and S9. In Figure 2 the five peaks in the O K-edge XAS spectra are marked, accordingly, a-b (due to a transition from O 1s to unoccupied O 2p–Ti 3d orbitals in an O_h_ crystal field, split into 531.5 eV (*t*_2g_) and 534.0 eV (e_g_) components) and d-f (540.8, 543.3, and 546.2 eV; O 1s to O 2p–Ti 4s,p transition), consistent with the presence of the rutile structure.^37,38^ Peak c can be assigned to a contribution from cobalt oxides when in the field of view.^39^ Here we analyze the differences in the normalized relative intensity of features (a) and (b) to provide insight into the local structural and electronic state of Ti. For example, we observe that the peak (a) in the spectra recorded for the fresh sample and shown in Figure 2a is much lower in intensity when compared to the peak (b) (*I*__eg__/*I*_t2g_ ≥ 1.30, Table S2). Furthermore, we determine the 10Dq splitting to be ∼2.3 eV (Table S2), as shown in Figure 2a (Co/Ti-1) which is much lower than the 2.6 eV shown in Figure 2b (Co/Ti-2), more typical of crystalline rutile.^38,40^ This relative decrease in peak (a) intensity^41^ and 10Dq^42^ has previously been ascribed to Ti^3+^ formation and^38,42,43^ the increased electron population in the Ti 3d t_2g_ state, reducing the dipole transition probability from the O 1s. The number of surface O_vac_ or Ti^3+^ can be correlated with 10Dq and the ratio of *I*__eg__/*I*_t_2g__; namely, the lower 10Dq or higher *I*__eg__/*I*_t_2g__, the more O_vac_ or Ti^3+^ ions are present on the surface. Focusing on the whole field of view (6 μm, including NPs), it was possible to perform the same analysis to identify regions within samples with variable O_vac_ (see Figure S4g). The O_vac_ are considered to be mainly distributed at the surface of the rutile TiO_2_^33,44^ in single- and double-cluster forms^45^ because a low-pressure air plasma treatment was used to create O_vac_. It is also possible that some O_vac_ are present in the bulk due to the calcination of TiO_2_ at 773 K,^46^ and these O_vac_ may migrate to the subsurface when at higher temperatures or with a change in the chemical potential.^47^

**Figure 3:**
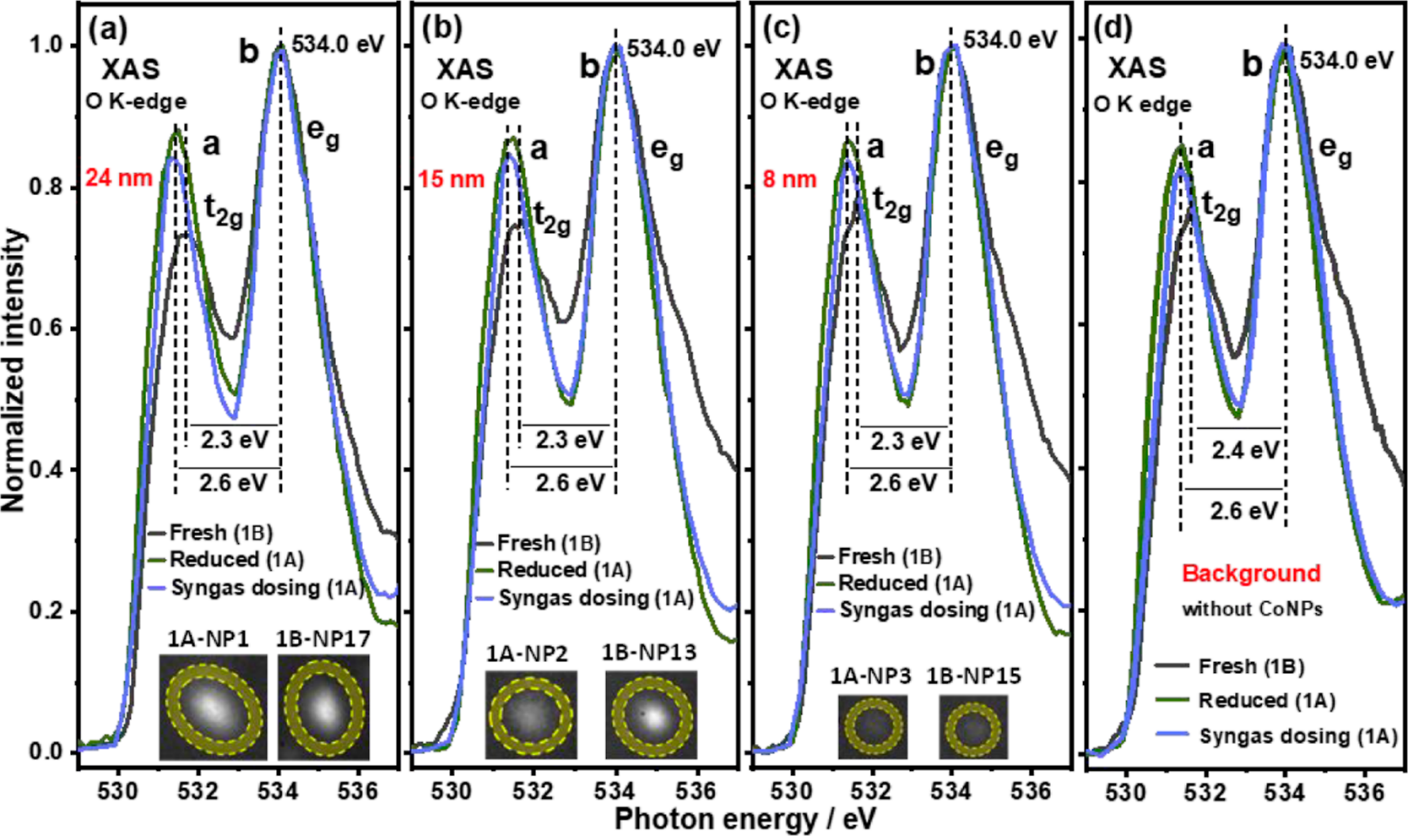
(a–c) Local XAS spectra of O K-edge in the defined periphery (shaded yellow ring) of the NPs at different stages of Co/Ti-1A/B. (d) XAS spectra differences of O K-edge in the pure TiO_2_ substrate for Co/Ti-1. All the spectra in (a–c) are normalized to the intensity of the e_g_ peak at 534 eV. The t_2g_ peak intensities are always lower than those observed for the e_g_ peaks, they also possess low splitting energy (10 Dq = 2.3 eV in Figure 3a–c fresh sample (Co/Ti-1B) in comparison to ∼ 2.7 eV of 10 Dq in rutile shown in Figure S4(42)) indicating the presence of O_vac_.

Further confirmation of the presence of a modified TiO_2_ surface can be gleaned from XPS which has previously been used to identify the presence of O_vac_/Ti-OH species, in particular by the observation of peaks at ∼531.2 and ∼532.5 eV.^48,49^ These data have, furthermore, been directly correlated with bulk techniques sensitive to spin state, namely EPR.^49,50^ As such, the presence of surface O_vac_ in our sample is confirmed by the presence of such O 1s peaks and by the signature Ti 2p XPS spectra shown in Figure S3b,c. From the fitted results of the O 1s spectra (Figure S3d), the O_vac_ in the fresh sample occupies 12.3% of the surface. XPS also allows us to determine that only 15.1% of these are Ti–OH species, possibly formed as a result of a reaction between surface O_vac_ and hydroxyl radicals (generated by air plasma)^49^ or hydration after exposure to air.^51^ Previous work has also demonstrated that the presence of O_vac_ often leads to the formation of undercoordinated Ti^3+^ species and that these species have a bigger role in the cobalt oxidation state than the presence of Ti–OH.^52^ Subsequently we also observe the presence of Ti^3+^ (6.7%) from the fitting of the Ti 2p XPS spectra in Figure S3c as well as evidence of Ti^3+^ in the Ti L_3,2_-edge XAS spectra (see Figure S10 and Table S3).^37,42,53^ We observe the Ti L-edge XAS spectra to be quite different from rutile; this is, at least in part, due to the difficulty of obtaining Ti spectra through the Co NPs. This prohibits a more detailed analysis of these data but by using Δ*E*_eg2-eg1_ as a guide to the ratio of Ti^3+^/Ti^4+^, it appears that the trends shown in Table S3 match broadly with those seen in [O_vac_] shown in Figures 3 and S4.

### Role of Oxygen Vacancies on Co NP Reduction and Their Behavior in H_2_ and Syngas

It is now possible to rationalize the impact of the O_vac_ on the stability of the various Co NPs based on the following standard electrode potentials: Ti^4+^ + e^–^ → Ti^3+^ (−0.56 V); Co^2+^ + 2e^–^ → Co (−0.28 V); and Co^3+^ + e^–^ → Co^2+^ (1.82 V), which allows us to determine that the potential for reduction/oxidation of Co/Ti is positive, that is, for Co^3+^ + Ti^3+^ → Co^2+^ + Ti^4+^ it is 2.38 V, and for Co^2+^ + 2Ti^3+^ → Co + 2Ti^4+^ it is 0.84 V. Hence the reduction of Co^3+^ to Co^2+^ and Co^2+^ to Co^0^ with surface O_vac_/Ti^3+^ would be spontaneous, occurring without the need to apply heat and/or reducing agents should the O_vac_/Ti^3+^ species be present in sufficient quantities. This can explain in particular why the edges of the Co NPs and the entirety of the small NPs in Co/Ti-1A (containing the greatest amount of O_vac_/Ti^3+^ at the edges based on high *I*_e_g__/*I*_t_2g__ and low 10Dq values in Table S2) are typically the most reduced. In contrast, the lack of surface O_vac_ in Co/Ti-2A renders the NPs stable as Co_3_O_4_ in the fresh sample (Figure S7a).

Treatment of the sample at 623 K in H_2_ leads to a decrease or “filling in” of the number of O_vac_ in region Co/Ti-1 as evidenced (in Figure 3a–c) by a change in the *I*_eg_/*I*_t_2g__, which decreases from > 1.30 to ∼ 1.14; that is peaks attributable to t_2g_ peaks were observed to increase in regions close to Co NPs whilst 10 Dq increases from ∼2.3 to ∼2.6 eV (Table S2). Note that Figure 3d, depicting the TiO_2_ background, undergoes the same changes indicating that the changes in O_vac_ occur in the entire sample. Strikingly, we observe an interesting correlation. In regions with higher [O_vac_] (e.g., 1A-NP1(24 nm) in Co/Ti-1A) the reduction of the edge of the NP is more pronounced than for NPs in regions with a low [O_vac_] (e.g., 2B-NP27(22 nm) in the Co/Ti-2), where *I*_e_g__/*I*_t_2g__ < 1 in Table S2. We attribute this disparity in Co/Ti-2 to the strong bonding interactions at the edge of Co NPs with the TiO_2_ surface.^23^ This also leads to differences in the degree of reduction of the centers of the NPs with those in region Co/Ti-1 being fully reduced for NPs < 15 nm (see Table 1), whereas in the region Co/Ti-2 only < 40% cobalt reduction is observed (see Table S1). The larger NPs in both samples are reduced to the lowest extent, particularly those in sample Co/Ti-2 present so much unreduced cobalt attributable to the high diffusion activation barrier. Furthermore, it appears that for all of the Co NPs in region Co/Ti-2, the centers of the NPs are more reduced than their edges (∼37 (center) versus ∼ 25% (edge) in 2B-NP29(15 nm)) see Table S1.

Syngas dosing and reaction at 493 K for 30 min then lead to a decrease in intensity of the t_2g_ peaks both nearby the Co NPs and in the TiO_2_ background in region Co/Ti-1, suggestive of the formation of new O_vac_ after reduction, although they are now fewer in number than what was observed in the fresh sample (Figure 3, Table S2). This is in contrast to the Co/Ti-2 sample, where the number of O_vac_ increases for all Co NPs after syngas treatment as well as the TiO_2_ background (Figure S11 and Table S2). Note though that overall, Co/Ti-1 always possesses a greater number of O_vac_ than Co/Ti-2. The increase in the number of O_vac_ in both samples is due to the greater reducing power of syngas than pure H_2_, even considering the differences in the temperature of the treatment (623 K for H_2_, 493 K for syngas).^26,54^ The differences in concentration of O_vac_ surrounding NPs in Co/Ti-1 and Co/Ti-2 are shown in Figure S12. No obvious size-dependent correlation between NPs and O_vac_ can be discerned, however.

**Figure 4:**
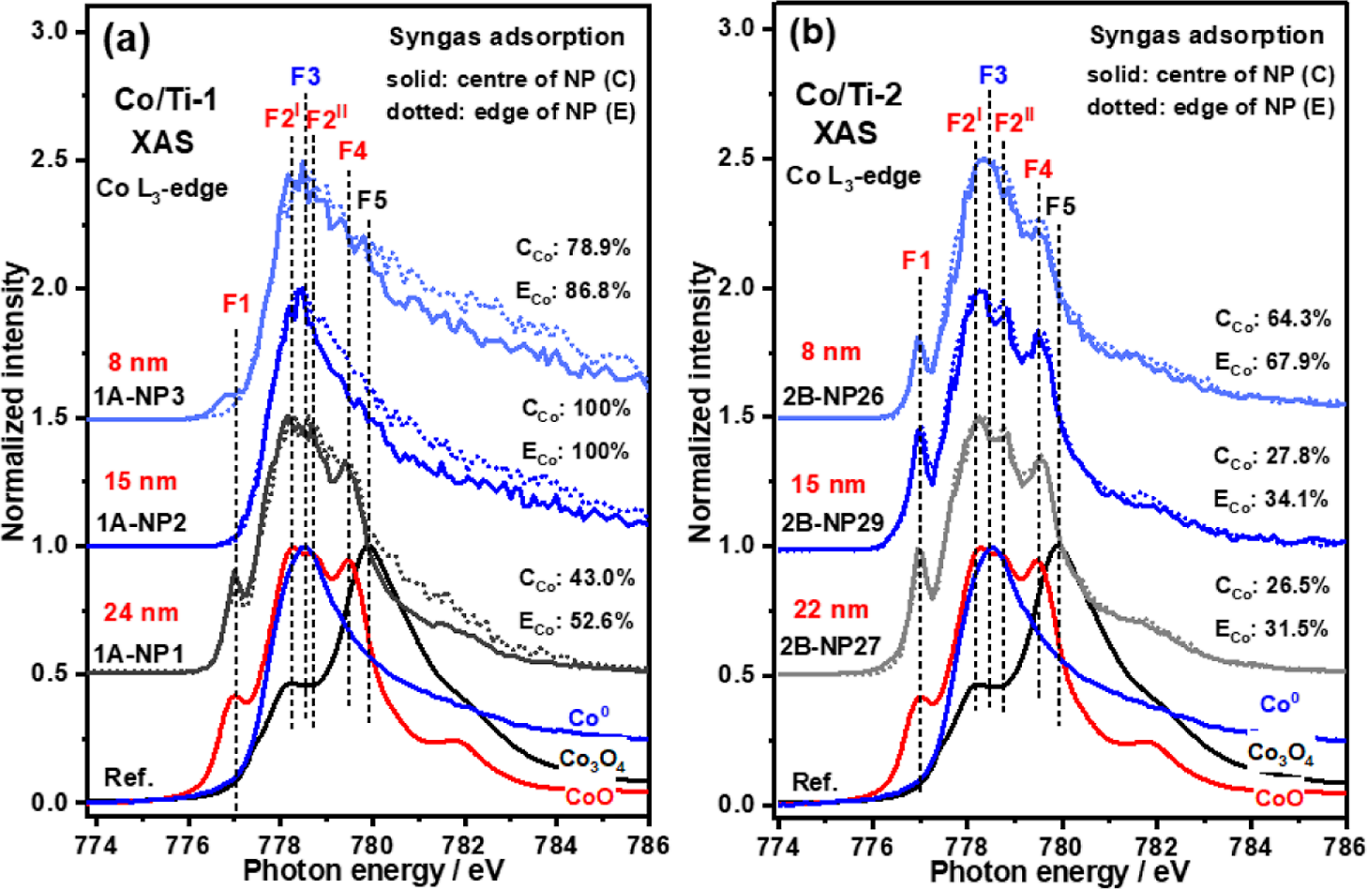
Co L_3_-edge XAS spectra after syngas adsorption in regions Co/Ti-1 (a) and Co/Ti-2 (b). O_vac_ on the TiO_2_ surface can prevent small Co NPs (<8 nm) from being oxidized but promote further reduction for big NPs (>15 nm).

XAS spectra of Co L_3_-edge after syngas treatment are displayed in Figure 4 for regions Co/Ti-1 and Co/Ti-2. Syngas exposure is seen to promote the further reduction of all NPs in region Co/Ti-2, but only the “big” NP in region Co/Ti-1. This region also witnessed partial oxidation of the smaller (8 nm) Co NP. The reoxidation of small Co NPs has been observed on many occasions^30^ and may be caused by the presence of more undercoordinated sites in small NPs, which can strongly bind species like CO.^30,55^ Alternatively, it has been observed that the by-product of the reaction, H_2_O as well as the support, can cause oxidation.^30,56^

**Scheme 1:**
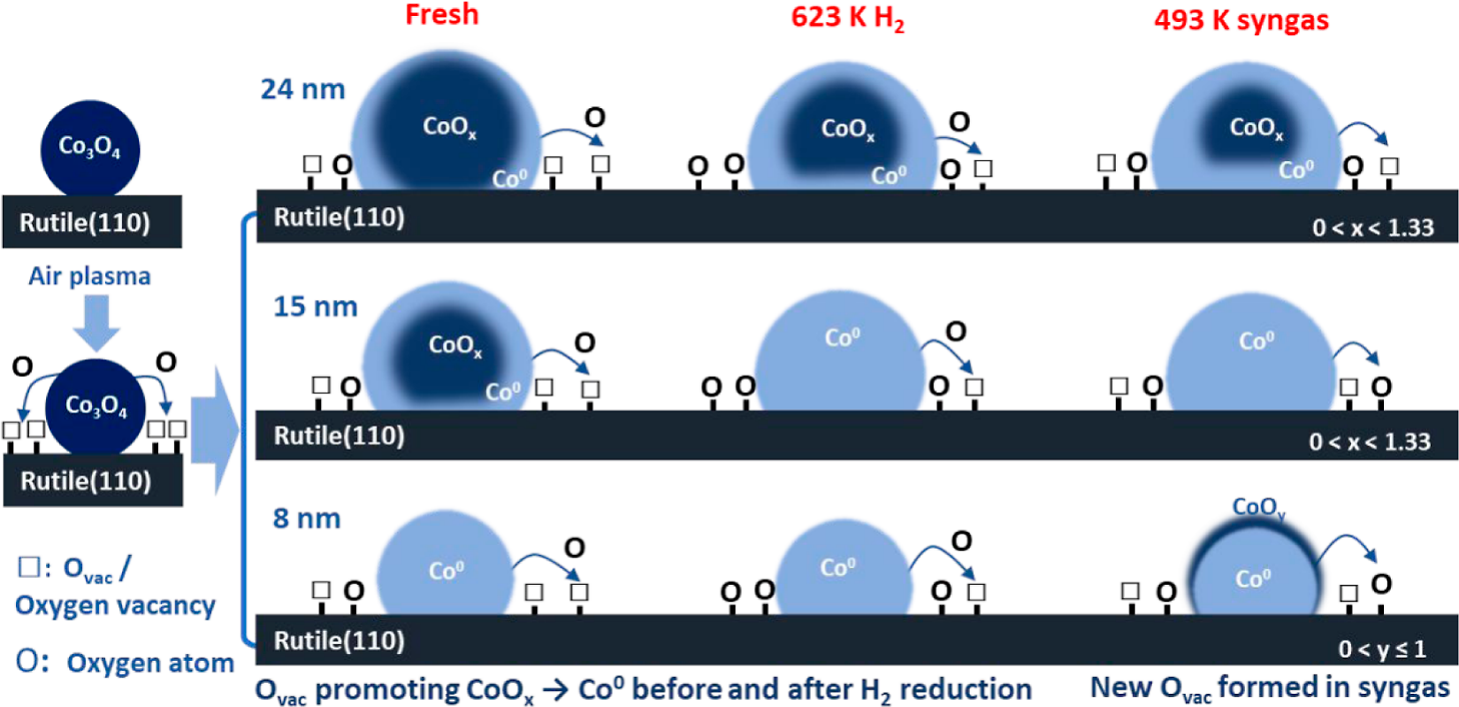
O_vac_ Promote Cobalt Oxide NPs (on Co/Ti-1) Reduction to CoO/Co^0^ Co_3_O_4_ NPs with the help of O_vac_ on rutile substrate surface can transform into CoO/Co^0^ in the fresh sample, and this process can be further enhanced by H_2_ reduction (623 K) and syngas adsorption (493 K). The detailed phase composition of different sized Co NPs at every step can be found in Table 1. Notably the smaller NPs, in particular the peripheries of the NPs, are more influenced by O_vac_ resulting in formation of more metallic cobalt. The corresponding O_vac_ changes are shown in Figure 3 and Table S2.

Comparing the spectra recorded at the edges and centers of the NPs, in both Co/Ti-1 and Co/Ti-2, it is noticeable that after syngas treatment the NPs edges are always more reduced than the centers (e.g., 43.0% Co^0^ at the center while 50.8% of Co^0^ at the edge in 1A-NP1(24 nm)). In contrast, for Co/Ti-2, the edges of NPs also possess higher amounts of metallic cobalt than the centers after syngas dosing (e.g., 2B-NP27(22 nm), 26.6 and 29.6% Co^0^ at the center and edge, respectively). From the O K-edge spectra evolution with syngas dosing (Figure 3, Table S2), we propose that the lower degree of oxidation seen at the edges of all but the 8 and 15 nm-sized particles seen in Co/Ti-1 has to do with the presence of formed O_vac_ on TiO_2_ acting as a sink for any oxygen-containing species adsorbed on the metallic cobalt, which then ensures more active sites for syngas adsorption and conversion.^12,26,27^ In Scheme 1 we depict the proposed effect of O_vac_ on the Co phase evolution of the three NPs profiled in region Co/Ti-1 and illustrate how their presence aids the formation and stability of metallic Co.

### Summary and Conclusions

Reducibility of metal NPs is a crucial aspect of the design of catalysts with improved performance for FTS and other important catalytic processes. Surface O_vac_ have been shown to enhance the extent of NP reduction, opening new pathways for the preparation of active and selective supported catalysts. Herein, a combination of *quasi in situ* X-ray photoemission electron microscopy (X-PEEM) and soft XAS has been used to investigate the promotional effect of O_vac_ on the reduction behavior of supported Co_3_O_4_ NPs, showing the effect on the reducibility of O_vac_—generated by air plasma treatment, to be clearly dependent on the size of the NPs. This is a particularly important finding considering FTS (as well as other structure-sensitive reactions), as there is a strong dependency between the catalytic performance and the size of Co NP.

Our results evidence that in regions where a larger number of O_vac_ (Co/Ti-1) are present, Co_3_O_4_ NPs are readily reduced to CoO/Co^0^ (i.e. even in the fresh sample and before the reduction treatment), illustrating the promotional effect of O_vac_. Notably, the extent of reduction is seen to be dependent on the size of the NPs, with smaller particles (NP < 8 nm) being more reduced than the larger ones. Further NP reduction is observed after H_2_ and syngas treatment, accompanied by the consumption of O_vac_ after H_2_ exposure. Conversely, in regions with a lower concentration of O_vac_ (Co/Ti-2), all NPs are seen to remain oxidized to some extent.

Interestingly, an increase in the number of O_vac_ is observed after the treatment in syngas for Co/Ti-2, coincident with an increase in the degree of NP reduction, with O_vac_ seen to be able to prevent the complete reoxidation of small Co NPs (<8 nm) during syngas exposure. When a sufficiently large number of O_vac_ are present, the reduction of medium-sized Co NPs (e.g., 12 nm) as well as their stabilization in the reduced state during syngas treatment is realized.

Therefore, our findings suggest that an introduction of O_vac_ on a sample support surface is a promising and potentially straightforward method to develop catalytic materials with a greater extent of reducibility^24,26^ and stability. O_vac_ creation may be achieved by both plasma treatment and treatment of the samples at high temperature in a reducing atmosphere, although with the former treatment the O_vac_ appear to be inhomogeneously distributed on the surface of TiO_2_. An added advantage of the plasma treatment is that there is a lower risk of deactivation of the metal NPs either by agglomeration or encapsulation that high-temperature H_2_ treatment has been shown to induce in the past.^57^ Our results do not allow us to determine whether the inhomogeneity observed in the samples would also occur if reduction had been performed with gases at high temperatures.

While it is not clear why different regions of the same sample should contain and retain different [O_vac_], it seems likely that this must be related to some difference in TiO_2_ structure. Indeed, it has been shown previously that the nature of the TiO_2_ polymorph affects the redox properties of the Co NPs.^58^ As an added bonus, O_vac_ have also been proposed to improve intrinsic reaction activity at the cobalt–titania interface by promoting the transformation of adsorbed oxygen-contained species.^27,59^ These results also indicate the value of studying 2D catalysts, particularly where the probing of the fundamentals of catalysis is concerned. We have shown here intimate insight into the influence of the properties of the support on the NP as a function of size under conditions akin to those found during the reaction. We have shown how this insight can be obtained by combining preparation methods for controlling particle size with *in situ* nano/micro-spectroscopy at multiple edges. This approach has been demonstrated to be very revealing for examining the correlation between particle size, their properties, and how these might influence catalytic performance.

## Experimental Section

5 g of tetraethylene glycol monododecyl (C_12_E_4_, Brij L4, Sigma-Aldrich) was added into 26.67 g of *n*-hexane (Sigma-Aldrich, 303 K water bath) and then stirred at 500 rpm for 2 h. After forming a colorless solution (reverse micelles formed), 960 mg of Co(NO_3_)_2_·6H_2_O (≥98%, Sigma-Aldrich, in 0.6 mL deionized water) was added and stirred for another 2 h under the same conditions, the solution turned transparent pink immediately. Then adding 25 wt % NH_3(aq)_ (0.9 g, Sigma-Aldrich), the solution turned from pink to green to cyan to dark green. >60 mL acetone was added to break the micelles and release Co(OH)_2_ NPs. The resultant precipitate was washed with acetone 3–5 times to fully remove C_12_ × 10^4^, dried at 373 K for 12 h, and calcined at 473 K for 5 h.^60^ The produced Co_3_O_4_ NPs were ∼18 nm in diameter. By decreasing the amount of cobalt nitrate hexahydrate added to 380 mg or 240 mg, ∼11 and ∼6 nm Co_3_O_4_ NPs could also be obtained (shown in Figure S1).

Co_3_O_4_ NPs (mixtures of 6, 11, and 18 nm NPs) were dispersed into ethanol using an ultrasonic bath (20 min), and then, the solution became dark black. After removing the very big NPs (because of agglomeration during preparation) by using centrifugation (8000 rpm, 5 min), the black solution became yellow. Before deposition, the TiO_2_(110, rutile) substrate (10 × 5 × 1 mm, purchased from Crystal GmbH) was calcined at 773 K for 6 h in a muffle furnace and then was cleaned in an ultrasonic bath by using acetone and isopropanol. Then, the yellow NP solution was deposited on the substrate using a dip-coater at room temperature at the speed of 5 mm/min. After drying at 473 K for 5 h, the prepared sample was treated in air plasma on a Diener Femto low-pressure plasma system model 1B2 to create O_vac_. The output power was 100 W and the pressure of the system was controlled at 0.3 mbar through the adjustment of airflow rate. The sample was treated with air plasma for 1 h at room temperature without additional heating. The distribution of NPs on the substrate is shown in Figure S2 AFM images.

The prepared NPs were dispersed into ethanol using an ultrasonic bath for 30 min and then around 10 drops of supernatant were deposited onto a copper TEM grid (mesh size 200) with a carbon film, dried at room temperature in air, and measured by a JEM2100 TEM 200 kV instrument. The NPs sizes were analyzed by ImageJ 1.52e.

The crystallinity of the prepared Co_3_O_4_ NPs was confirmed by using a Rigaku Smartlab XRD instrument (CuKα1, 45 kV, 2θ 20–70°, step 0.01°, speed 0.2 s/°) with fixed divergence slits at the ISIS neutron and muon light source. The average NP size was estimated by the Scherrer equation.

The supported NPs after air plasma etching were measured in air by a Bruker Veeco MultiMode V atomic force microscope at Diamond Light Source (DLS) in the tapping mode at a scan rate of 1 Hz (RT, 1 atm; cantilever: Bruker RTESPA-300). From the obtained AFM images, the Co NPs size (height) and distribution could be analyzed by Gwyddion 2.49.

XPS measurements were performed on a Thermo Fisher Scientific NEXSA spectrometer at HarwellXPS. Samples were analyzed using a micro-focused monochromatic Al X-ray source (72 W) over an area of approximately 400 μm. Data were recorded at pass energies of 200 eV for survey scans and 50 eV for high-resolution scans with 1 eV and 0.1 eV step sizes. Charge neutralization of the sample was achieved using a combination of both low-energy electrons and argon ions. All the samples were measured under a vacuum of 10^–9^ mbar and room temperature. The obtained data were analyzed by CasaXPS 2.3.19PR1.0. The binding energy of Co 2p and O 1s was calibrated by using C 1s (284.8 eV).

X-ray absorption spectroscopy and photoemission microscopy were together carried out at I06 at DLS using an X-PEEM equipped with an energy analyzer. The beamline provided high brilliance X-rays in the energy range of 130–1500 eV. In order to obtain elemental contrast X-PEEM images (field of view 6 μm), images at cobalt L_3,2_-edge absorption edge and below the absorption edge were recorded sequentially by using the total electron yield mode. Also, the O K-edge and Ti L_3,2_-edge were recorded under the same conditions as Co L_3,2_-edge. The bright spots may correspond to individual Co NPs but have to be confirmed by X-ray absorption spectroscopy. The base pressure in the X-PEEM was 1 × 10^–9^ mbar. Dosage of hydrogen and syngas was controlled at a pressure of 1 × 10^–6^ mbar in a prechamber. Hydrogen reduction was conducted at 623 K for 3 h, while syngas was performed at 493 K for 30 min, respectively. The pure powdered Co_3_O_4_, CoO, and Co^0^ references (50–100 nm, measured at ISISS beamline, BESSY) were provided by Dr. Liping Zhong at EPFL. All the data were analyzed by the software of ImageJ 1.52e and Origin Pro 2019, and the spectra of Co L_3_-edge below 776 eV were smoothed. The linear combination fitting was done by using Athena 0.9.26 software.^62^ All the spectra were subtracted by TiO_2_ background and normalized as in the previous work before fitting.^61^ O_vac_ presenting on the TiO_2_ surface were rando; therefore, X-PEEM regions with abundant O_vac_ were denoted as Co/Ti-1A or 1B, while the regions that lack O_vac_ were Co/Ti-2A and 2B.

To correlate the real size of focused Co NPs in the X-PEEM images, the samples after X-PEEM measurement were imaged by using a Zeiss Crossbeam 550 XL equipped with a Gemini II FE-SEM column. The images were acquired at 2 kV 35pA with a pixel dwell time of 50 ns, scan speed of 0, line averaging of 100, and a store resolution of 3072 × 2304 pixels. Lastly, the obtained images were analyzed by using ImageJ 1.52e.

## Supporting Information Available

Additional sample characterization data; supplementary spectra; exemplar fits to the XAS data (PDF)

## Notes

Raw data from this publication are available from the following DOI: 10.5522/04/20171153.
