# Supplementary material for: Resolving the Effect of Oxygen Vacancies on Co Nanostructures Using Soft XAS/X-PEEM
Source: ACS Catal. 2022 Jul 14;12(15):9125–34. doi: 10.1021/acscatal.2c00611 (PMC9361287; doi:10.1021/acscatal.2c00611)
Supplement: Supplementary file 1 — cs2c00611_si_001.pdf [file cs2c00611_si_001.pdf]

Supporting information

Resolving the effect of oxygen vacancies on Co nanostructures using soft XAS/X-PEEM

Chengwu Qiu[1, 2], Yaroslav Odarchenko[1, 2, †], Qingwei Meng[3], Shaojun Xu[2, 4], Ines Lezcano-Gonzalez[1, 2], Paul Olalde-Velasco[5], Francesco Maccherozzi[5], Laura Zanetti-Domingues[2], Marisa Martin-Fernandez[2], Andrew M. Beale[1, 2] *

1 Department of Chemistry, University College London, 20 Gordon Street, London, WC1H 0AJ, UK

2 Research Complex at Harwell (RCaH), Harwell, Didcot, Oxfordshire, OX11 0FA, UK

3 School of Chemical Engineering and Light Industry, Guangdong University of Technology, Guangzhou 510006, China

4 Cardiff Catalysis Institute, School of Chemistry, Cardiff University, Cardiff, CF10 3AT, UK

5 Diamond Light Source, Harwell, Didcot, Oxfordshire, OX11 0DE, UK

**corresponding author: Andrew.Beale@ucl.ac.uk*

Table of contents

Figure S1. TEM image and XRD patterns showing spherical shape of Co_3O_4 nanoparticles used for Co/TiO_2 preparation.

Figure S2. AFM images and histograms of nanoparticle distribution for Co/TiO_2 catalyst before X-PEEM experiment.

Figure S3. Co 2p, O 1s and Ti 2p spectra of Co/TiO_2 catalyst before and after X-PEEM experiment.

Figure S4. X-PEEM images with XAS O K-edge spectra and their corresponding histograms of nanoparticle distribution for Co/TiO_2.

Figure S5. Correlation the size of Co NPs in X-PEEM images using high resolution SEM.

Figure S6. An example (1A-NP2-fresh) showing the differences of **.** Co L_3-edge XAS spectra between edge and centre of a NP.

Table S1. Linear combination fitting results from Co L_3-edge XAS spectra of Co/Ti-2.

Figure S7. Co L_3-edge XAS spectra of NPs before (a) and after reduction (a) in the Co/Ti-2 region.

Table S2. Peak parameters of O K-edge XAS spectra of Co/Ti-1 and Co/Ti-2 after H_2 reduction and syngas treatment.

Figure S8. Two examples of Linear combination fitting results for Co L_3-edge XAS spectra (screenshot).

Figure S9. XAS spectra of O K edge in Co/Ti-1 and Co/Ti-2 before and after H_2/syngas treatments.

Figure S10. Ti $L_{3,2}$-edge of Co/TiO_2 in fresh sample.

Table S3. Peak fitting parameters of Ti $L_{3,2}$-edge XAS spectra of Co/Ti-1 and Co/Ti-2 after H_2 reduction and syngas treatment.

Figure S11. Local XAS spectra of O K-edge in defined periphery (yellow shadow ring) of the NPs at different stages of Co/Ti-2A/B.

Figure S12. Size-correlated XAS spectra of O K-edge at the periphery of NPs after reduction and syngas treatment.

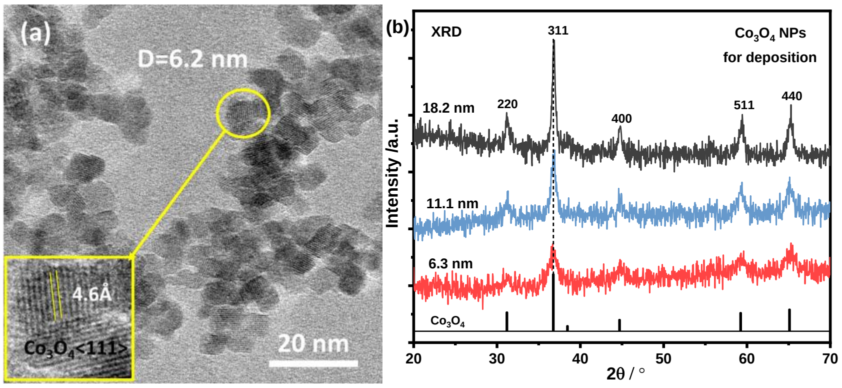

Figure S1. TEM image (a) and XRD patterns (b) showing spherical shape of Co_3O_4 nanoparticles used for Co/TiO_2 preparation.

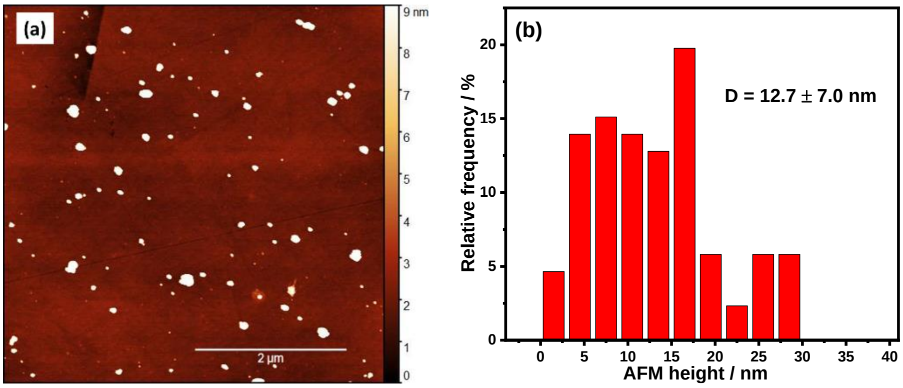

Figure S2. AFM images (a) and corresponding histograms (b) of nanoparticle distribution for Co/TiO_2 catalyst before X-PEEM experiment. The different heights of nanoparticles shown in Figure S2a are in line with the different sizes of Co_3O_4 nanoparticles.

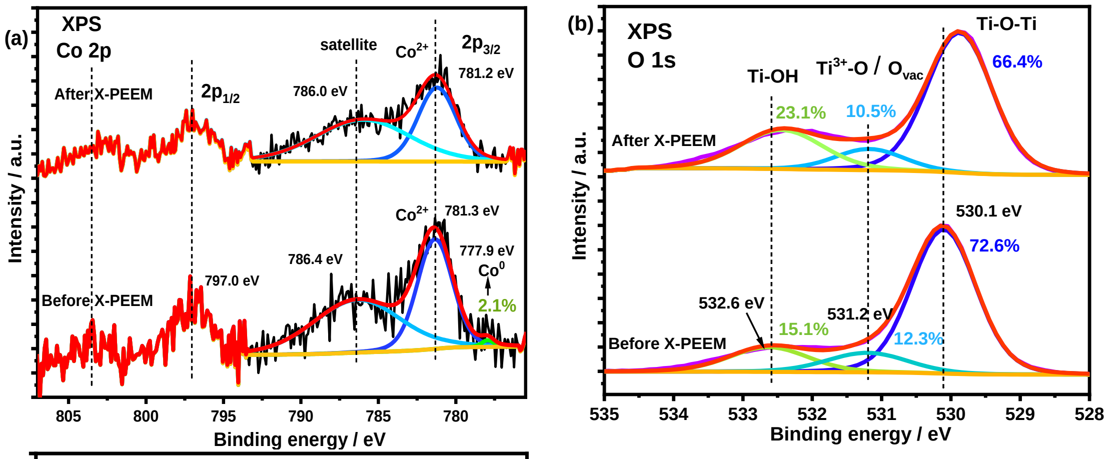

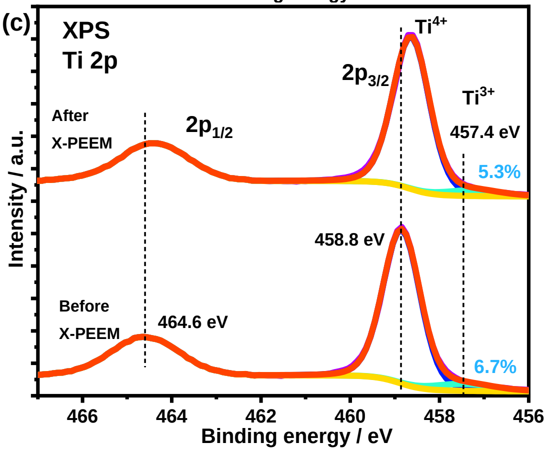

(d) The fitted results from the XPS spectra in (a)-(c).

Ratio/%	Co 2p		O 1s			Ti 2p	
	Co^0	Co^{2+}	Ti-O-Ti	O_{vac}	Ti-OH	Ti^{4+}	Ti^{3+}
Before X-PEEM	2.1	97.9	72.6	12.3	15.1	93.3	6.7
After X-PEEM	0	100	66.4	10.5	23.1	94.7	5.3

Figure S3. XPS Co 2p (a), O 1s (b) and Ti 2p (c) spectra of Co/TiO_2 before and after X-PEEM experiment. (d) Fitted results of Co 2p, O1s and Ti 2p XPS spectra from (a)-(c). Co^{2+} (CoO, ~781.3 eV) as main component before and after X-PEEM is shown in Figure S3a but small amount of metallic cobalt (2.1 %) is detected in the fresh sample.[1] The content of O_{vac} decreased by 1.8 % in the X-PEEM experiment while the Ti-OH increased by 8.0 %.[2,3] Ti^{3+} is detected before and after X-PEEM but quenched by 1.4 % in the experiment. [2,3,4]

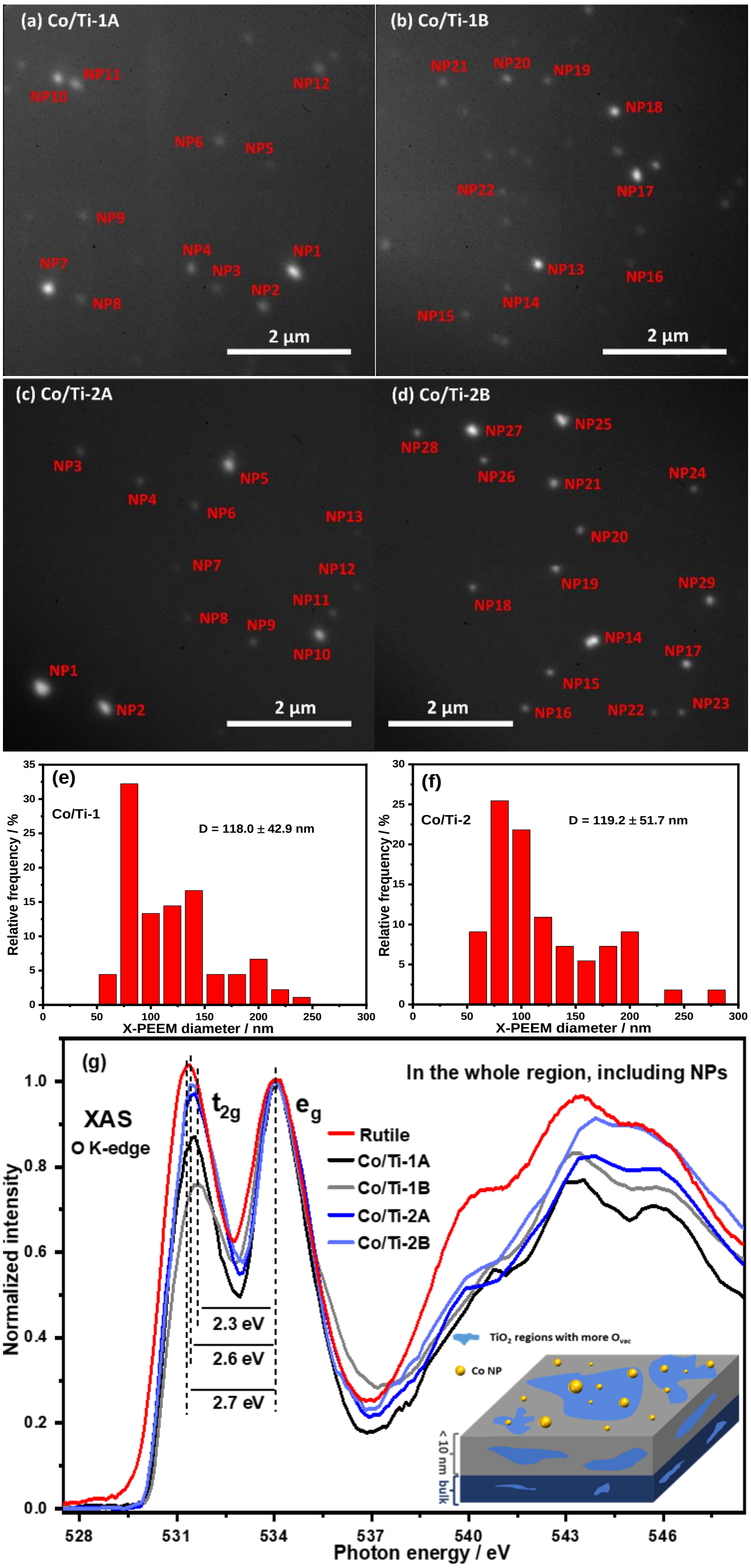

Figure S4. X-PEEM images (a-d, recorded at 778.3 eV, field of view 6 μm) showing nanoparticle distribution (e, f) in different regions (Co/Ti-1 or Co/Ti-2) for sample Co/TiO_2. The brightness or diameter reflects the size of labelled individual nanoparticles. (g) The XAS O K-edge spectra in the whole region of (a-d) with an insert image showing the envisaged O_{vac} distribution in rutile. Obviously, t_{2g} intensity and the splitting energy of t_{2g}-e_g in region Co/Ti-1 are always lower than that in region Co/Ti-2, meaning the concentration of O_{vac} in Co/Ti-1 is higher than in Co/Ti-2. All the O K-edge spectra are normalized to the intensity of the e_g peaks.

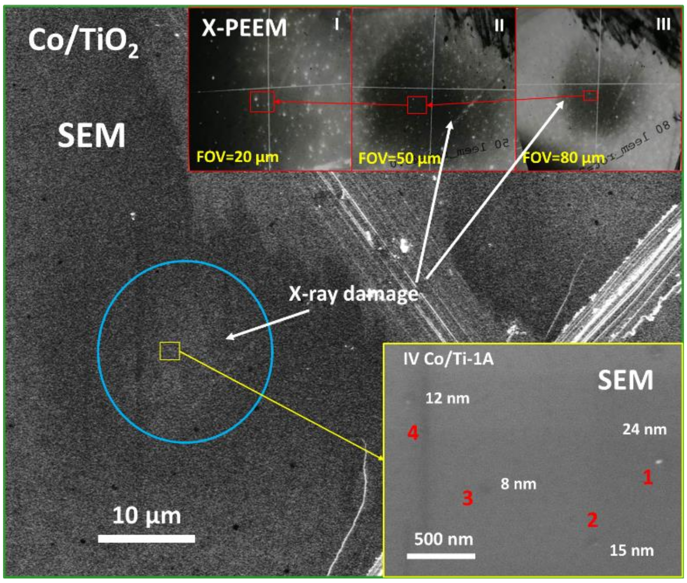

Figure S5. Determination of Co NPs in X-PEEM images using high resolution SEM, confirming the absolute size of the NPs to be 10 times smaller than the size determined from the X-PEEM images (Figure S4). Inserts: (I-III) X-PEEM images; (IV) SEM images.

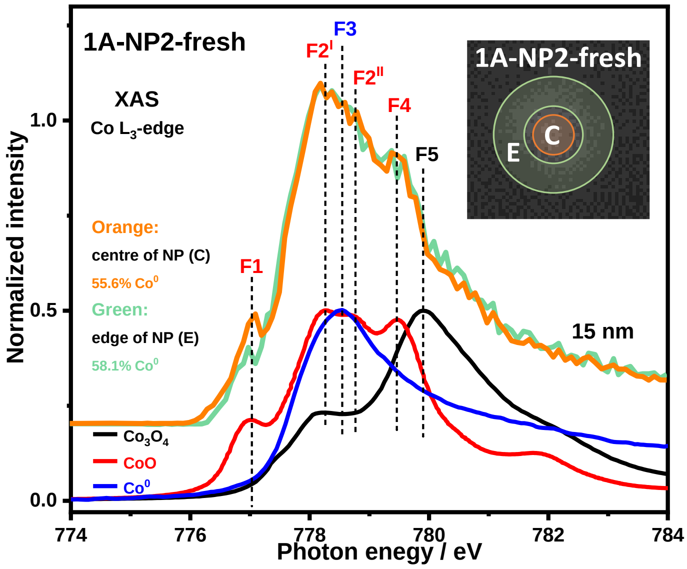

Figure S6. An example (1A-NP2-fresh) showing the differences of **.** Co L_3-edge XAS spectra between edge and centre of a NP. The insert image shows the definition of edge and centre of a NP.

Table S1. Linear combination fitting results from Co L_3-edge XAS spectra of NPs in region Co/Ti-2. The edge and entre definition are similar to Figure 1a and S8. The numbers in the parenthesis are the uncertainty errors in the fitting. Since the XAS spectra are noisy, some fitting errors are large, and in those cases the reliability of the analysis is lower.

Spectrum		Size/ nm	Co^0/%	CoO/%	Co_3O_4/%	R-factor	Reduced Chi-square
Fresh	2A-NP2-centre	23	0.8(5.0)	25.0(8.1)	74.2(2.4)	0.02212	0.002866
	2A-NP2-edge		0(4.8)	25.0(7.0)	75.0(2.3)	0.01951	0.002612
	2A-NP10-centre	16	6.2(0.7)	15.3(1.8)	78.5(2.5)	0.00874	0.001058
	2A-NP10-edge		5.7(0.5)	13.8(4.7)	80.5(1.0)	0.00430	0.000544
	2A-NP6-centre	8	9.7(9.7)	40.0(1.7)	50.3(1.7)	0.01938	0.002110
	2A-NP6-edge		8.2(9.7)	45.0(1.8)	46.8(1.8)	0.02230	0.002272
Reduced	2B-NP27-centre	22	29.6(8.3)	70.4(6.2)	0(1.7)	0.02303	0.003104
	2B-NP27-edge		26.6(5.9)	72.9(6.9)	0.5(9.1)	0.02667	0.003838
	2B-NP29-centre	15	36.9(5.9)	53.5(5.4)	9.6(8.1)	0.02903	0.003713
	2B-NP29-edge		25.0(6.0)	71.9(5.5)	3.1(8.1)	0.02655	0.003766
	2B-NP26-centre	8	41.9(9.5)	58.1(6.4)	0(2.1)	0.03375	0.004467
	2B-NP26-edge		31.4(8.2)	68.6(6.2)	0(1.8)	0.02219	0.003131
syngas adsorption	2B-NP27-centre	22	26.5(5.3)	67.6(6.0)	6.0(2.9)	0.02174	0.002953
	2B-NP27-edge		31.5(5.2)	62.9(5.9)	5.6(2.8)	0.01911	0.002789
	2B-NP29-centre	15	27.8(5.7)	67.5(6.4)	4.7(3.1)	0.02351	0.003415
	2B-NP29-edge		34.1(8.1)	64.0(5.3)	1.9(2.5)	0.03603	0.003829
	2B-NP26-centre	8	64.3(5.4)	35.7(3.5)	0(1.6)	0.01611	0.001589
	2B-NP26-edge		67.9(6.9)	32.1(4.9)	0(0.7)	0.03272	0.003077

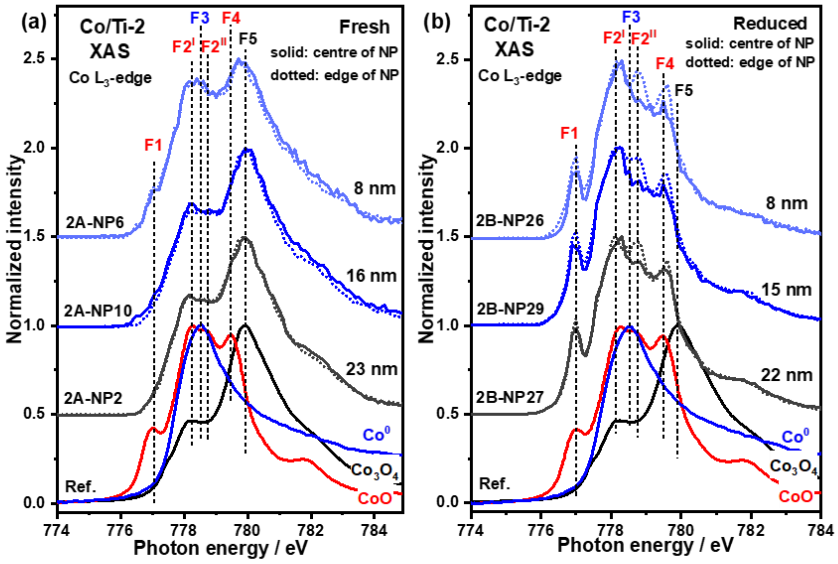

Figure S7. Co L_3-edge XAS spectra of NPs before (a) and after reduction (a) in the Co/Ti-2 region. Mainly Co_3O_4 is observed in fresh catalyst based on the XAS spectra in (a). Co NPs are not fully reduced but the centres in all size NPs are easy to reduce.

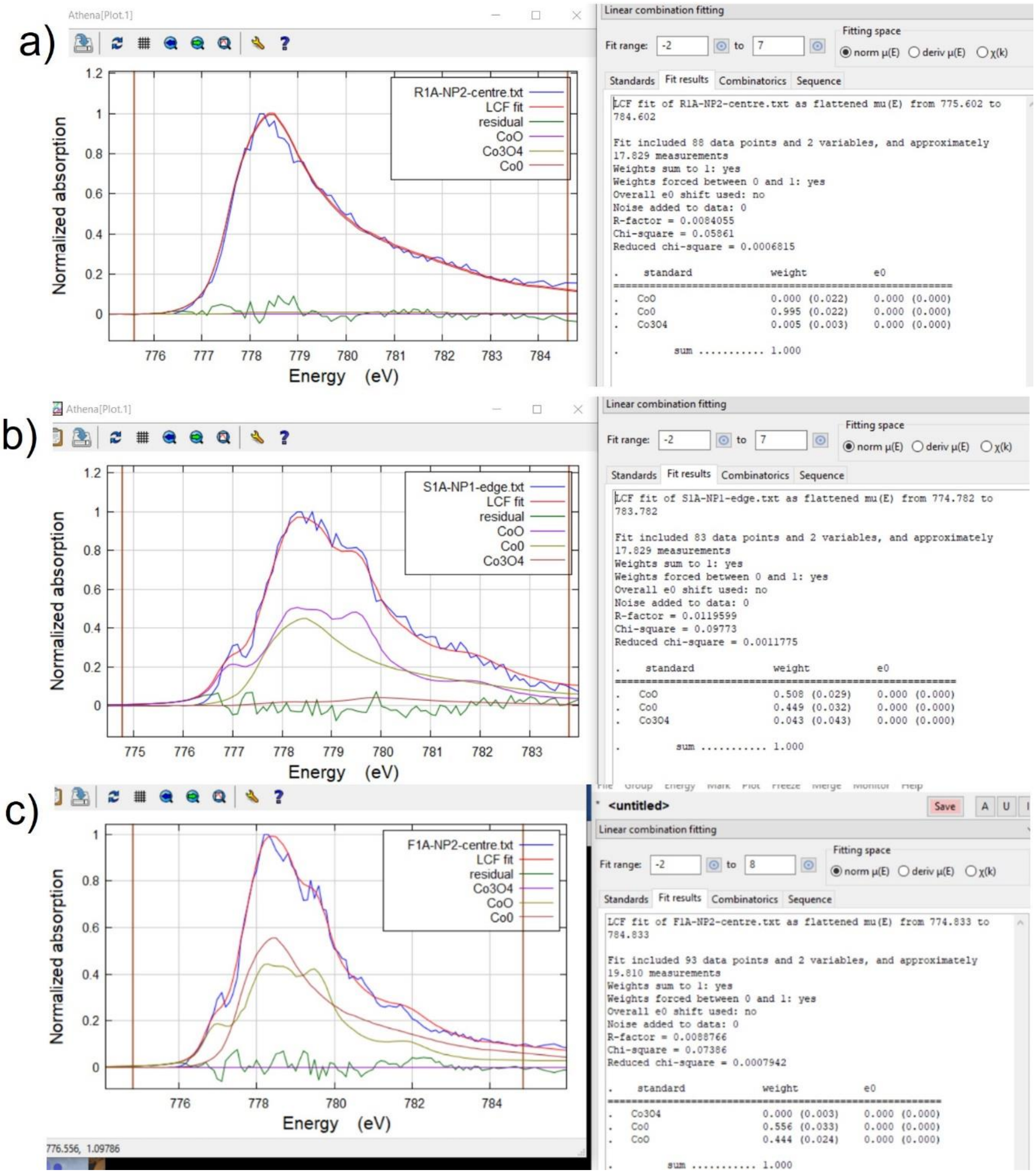

Figure S8. Three examples of linear combination fitting (LCF) results for Co L_3-edge XAS spectra (screenshots). (a) Reduced 1A-NP2-centre; (b) Syngas adsorbed 1A-NP1-edge; c) fresh F1A-NP2-centre.

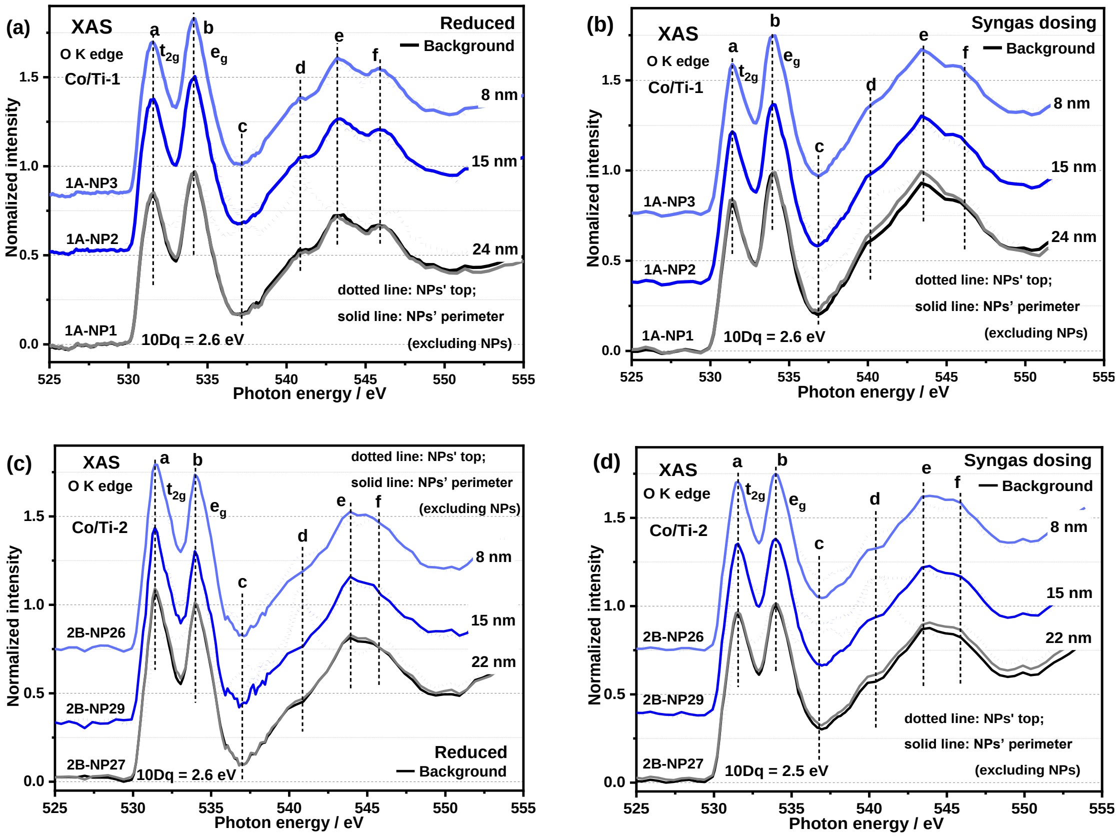

Figure S9. XAS spectra of O K edge in regions Co/Ti-1 and Co/Ti-2 after H_2/syngas treatments. All the spectra in a-d are normalized to the intensity of the e_g peaks. Note the strong feature c (dotted line) in a-d are attributed to the contributions from cobalt oxide.

Table S2. Peak parameters of O K-edge XAS spectra of NPs in regions Co/Ti-1 and Co/Ti-2 after treatment in H_2 and syngas.

O K edge							
Spectrum		Position/separation (eV)			Intensity	Size / nm
		t_{2g}	e_g	$10Dq/\Delta E_{eg-t2g}$	I_{eg}/I_{t2g}	
Fresh	1B-NP17	531.68	534.02	2.34	1.34	21
	1B-NP13	531.65	533.96	2.31	1.31	16
	1B-NP15	531.64	533.96	2.32	1.30	8
	1B-background	531.63	534.01	2.38	1.32	-
	2A-NP2	534.41	534.05	2.64	0.95	23
	2A-NP10	531.48	534.04	2.56	0.96	16
	2A-NP6	531.53	534.06	2.53	1.00	8
	2A-background	531.50	534.05	2.55	1.00	-
Reduced	1A-NP1	531.56	534.17	2.61	1.14	24
	1A-NP2	531.52	534.13	2.61	1.14	15
	1A-NP3	531.51	534.13	2.62	1.15	8
	1A-background	531.33	533.93	2.60	1.18	-
	2B-NP27	531.43	534.00	2.57	0.92	22
	2B-NP29	531.55	534.14	2.59	0.91	15
	2B-NP26	531.47	534.02	2.55	0.94	8
	2B-background	531.47	534.02	2.55	0.95	-
syngas adsorption	1A-NP1	531.43	534.00	2.57	1.18	24
	1A-NP2	531.41	534.00	2.59	1.18	15
	1A-NP3	531.42	533.96	2.54	1.19	8
	1A-background	531.38	533.95	2.57	1.22	-
	2B-NP27	531.55	533.98	2.43	1.05	22
	2B-NP29	531.50	533.98	2.48	1.04	15
	2B-NP26	531.52	533.97	2.45	1.05	8
	2B-background	531.63	534.05	2.42	1.05	-

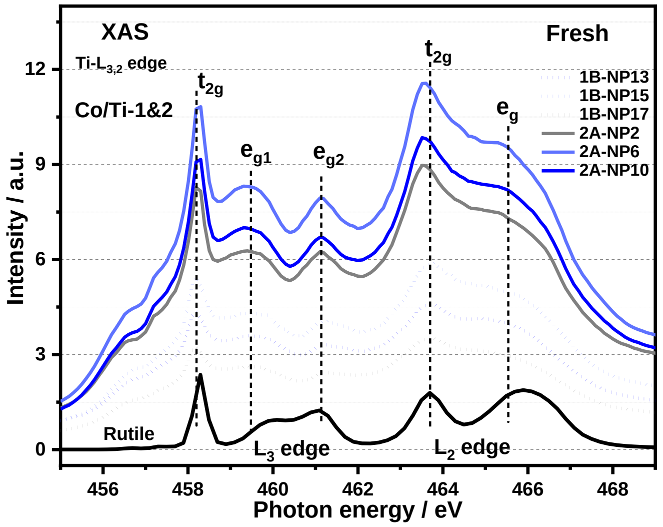

Figure S10. Ti $L_{3,2}$-edge of Co/TiO_2 in the fresh sample. The spectra were extracted from the top of the corresponding NPs in X-PEEM images.

Table S3. Peak fitting (using Lorentz) parameters of Ti $L_{3,2}$-edge XAS spectra of Co/Ti-1 and Co/Ti-2 after treatment in H_2 and syngas. The stronger e_g energy splitting reflect the numerous O_{vac} in Co/Ti-1.

Spectrum		Ti L3 (eV)					Ti L2 (eV)		Size / nm
		t_{2g}	e_{g1}	e_{g2}	$\Delta E_{eg2-eg1}$	I_{eg1}/I_{eg2}	t_{2g}	e_g	
Fresh	1B-NP17	458.18	459.43	461.38	1.95	2.03	463.69	465.67	21
	1B-NP13	458.18	459.48	461.39	1.91	2.00	463.67	465.62	16
	1B-NP15	458.19	459.47	461.33	1.86	1.88	463.72	465.73	8
	2A-NP2	458.21	459.38	461.18	1.80	1.51	463.61	465.65	23
	2A-NP10	458.23	459.39	461.19	1.80	1.61	463.62	465.68	16
	2A-NP6	458.23	459.39	461.20	1.81	1.64	463.53	465.68	8
Reduced	1A-NP1	458.08	459.73	460.99	1.26	1.45	463.56	465.78	24
	1A-NP2	458.07	459.73	460.98	1.25	1.62	463.56	465.75	15
	1A-NP3	458.06	459.71	460.99	1.28	1.57	463.56	465.75	8
	2B-NP27	458.04	459.23	461.00	1.77	1.75	463.47	465.35	22
	2B-NP29	458.01	459.20	460.99	1.79	1.70	463.44	465.35	15
	2B-NP26	458.03	459.20	461.01	1.81	1.75	463.46	465.34	8
syngas adsorption	1A-NP1	457.95	459.46	460.79	1.33	1.65	463.41	465.57	24
	1A-NP2	457.94	459.46	460.80	1.34	1.85	463.39	465.52	15
	1A-NP3	457.94	459.45	460.81	1.37	1.78	463.39	465.53	8
	2B-NP27	457.99	459.14	461.10	1.96	2.74	463.46	465.30	22
	2B-NP29	457.97	459.13	461.07	1.94	2.13	463.42	465.29	15
	2B-NP26	457.98	459.13	461.10	1.97	2.64	463.45	465.29	8
reference	TiO_2 (rutile)	458.57	460.17	461.30	1.13	0.55	463.96	466.29	-
	Ti_2O_3	457.44	459.02	460.47	1.45	1.02	464.58	466.06	-

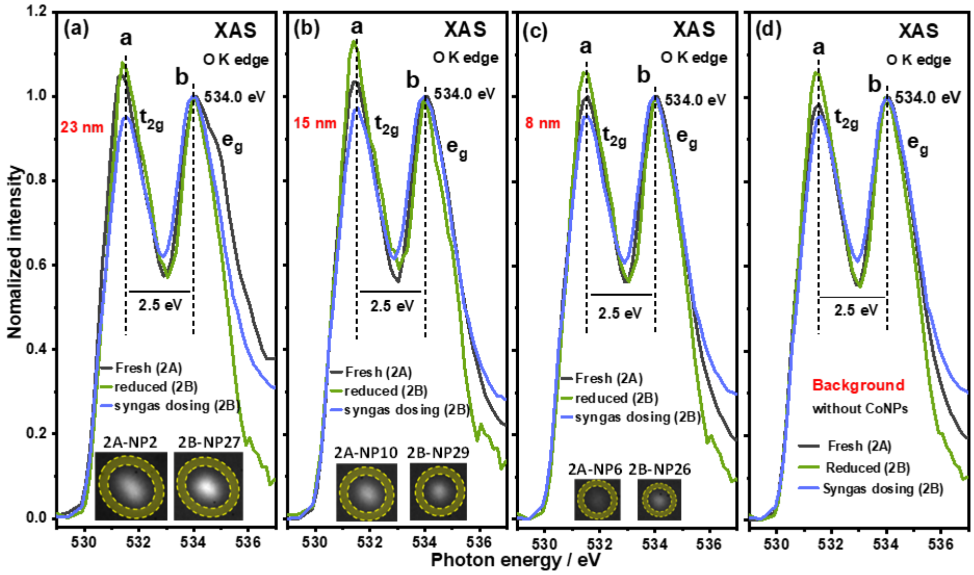

Figure S11. (a-c) Local XAS spectra of O K-edge in defined periphery (yellow shadow ring) of the NPs at different stages of Co/Ti-2A/B. (d) XAS spectra differences of O K-edge in the pure TiO_2 backgrounds for Co/Ti-2. All the spectra in a-c are normalized to 1 through e_g peaks. The t_{2g} intensities of O K-edge are significantly decreased after syngas dosing, meaning the formation of new O_{vac}.

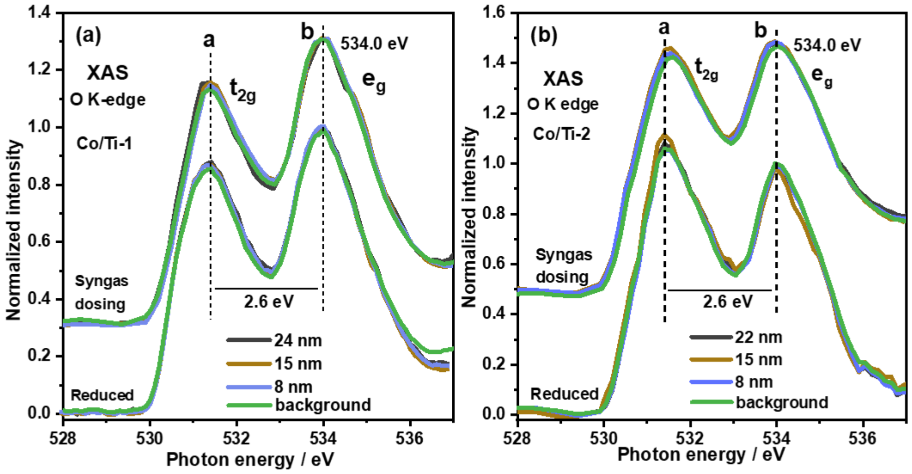

Figure S12. XAS spectra differences of O K-edge in the periphery of NPs and the pure TiO_2 backgrounds for Co/Ti-1 (a) and Co/Ti-2 (b) during various treatments. All the spectra in a-c are normalized to the intensity of the e_g peaks.

References

(1) Lukashuk, L.; Föttinger, K.; Kolar, E.; Rameshan, C.; Teschner, D.; Hävecker, M.; Knop-Gericke, A.; Yigit, N.; Li, H.; McDermott, E.; Stöger-Pollach, M.; Rupprechter, G. Operando XAS and NAP-XPS Studies of Preferential CO Oxidation on Co_3O_4 and CeO_2-Co_3O_4 Catalysts. *J. Catal.* **2016**, *344*, 1–15.

(2) Zhao, J.; Zhang, M.; Wan, S.; Yang, Z.; Hwang, C. S. Highly Flexible Resistive Switching Memory Based on the Electronic Switching Mechanism in the Al/TiO_2/Al/Polyimide Structure. *ACS Appl. Mater. Interfaces* **2018**, *10* (2), 1828–1835.

(3) Jedsukontorn, T.; Ueno, T.; Saito, N.; Hunsom, M. Facile Preparation of Defective Black TiO_2 through the Solution Plasma Process: Effect of Parametric Changes for Plasma Discharge on Its Structural and Optical Properties. *J. Alloys Compd.* **2017**, *726*, 567–577.

(4) Sasinska, A.; Singh, T.; Wang, S.; Mathur, S.; Kraehnert, R. Enhanced Photocatalytic Performance in Atomic Layer Deposition Grown TiO_2 Thin Films via Hydrogen Plasma Treatment. *J. Vac. Sci. Technol. A Vacuum, Surfaces, Film.* **2015**, *33* (1), 01A152.
